# Cellular and Molecular Mechanisms Underlying Non-Pharmaceutical Ischemic Stroke Therapy in Aged Subjects

**DOI:** 10.3390/ijms19010099

**Published:** 2017-12-29

**Authors:** Raluca Elena Sandu, Danut Dumbrava, Roxana Surugiu, Daniela-Gabriela Glavan, Andrei Gresita, Eugen Bogdan Petcu

**Affiliations:** 1 Department of Functional Sciences, Center of Clinical and Experimental Medicine, University of Medicine and Pharmacy of Craiova, Craiova 200349, Romania; danutdumbrava@gmail.com (D.D.); roxana.surugiu07@gmail.com (R.S.); Danaglavan@gmail.com (D.-G.G.); gresitaandrei@gmail.com (A.G.); 2 Gold Coast Campus, School of Medicine, Griffith University, Southport 4222, Australia; e.petcu@griffith.edu.au

**Keywords:** ageing, cerebral ischemia, obesity, inflammation, hypothermia, calorie restriction

## Abstract

The incidence of ischemic stroke in humans increases exponentially above 70 years both in men and women. Comorbidities like diabetes, arterial hypertension or co-morbidity factors such as hypercholesterolemia, obesity and body fat distribution as well as fat-rich diet and physical inactivity are common in elderly persons and are associated with higher risk of stroke, increased mortality and disability. Obesity could represent a state of chronic inflammation that can be prevented to some extent by non-pharmaceutical interventions such as calorie restriction and hypothermia. Indeed, recent results suggest that H_2_S-induced hypothermia in aged, overweight rats could have a higher probability of success in treating stroke as compared to other monotherapies, by reducing post-stroke brain inflammation. Likewise, it was recently reported that weight reduction prior to stroke, in aged, overweight rats induced by caloric restriction, led to an early re-gain of weight and a significant improvement in recovery of complex sensorimotor skills, cutaneous sensitivity, or spatial memory. Conclusion: animal models of stroke done in young animals ignore age-associated comorbidities and may explain, at least in part, the unsuccessful bench-to-bedside translation of neuroprotective strategies for ischemic stroke in aged subjects.

## 1. Introduction

The aging of the population, particularly in western societies, as well as the increasing prevalence of underlying risk factors results in a dramatic increase in the burden of cerebrovascular disorders such as stroke and vascular dementia. In aged humans, the incidence of stroke increases significantly with age both in men and women. In demographically developed countries stroke ranks 2nd in Europe, and 3rd in the United States and Canada reflecting the aged structure of western societies [1]. The average age at which stroke occurs is around 73 years, and it is the most frequent cause of permanent disability worldwide. In western countries stroke in humans is associated with high societal costs including costs for employers, families, social networks and caregivers [2]. 

## 2. Age Is the Principal Risk Factor for Ischemic Stroke

The incidence of stroke increases significantly with age both in men and women with incidence rates accelerating exponentially above 70 years [3]. However, there are gender differences in the incidence by age subgroups. Men aged up to 75 years old are more likely to be hit by stroke than women. The risk to have a stroke then becomes higher in women than men aged 85 years or older [3]. This may be attributed to sex-related differences in life expectancy of women and the development of age-related atherosclerosis. It should be noted that the age-associated decline in functional reserve is most pronounced after the age of 85, and implies an impaired response to stressors and illnesses. Importantly, age-associated changes show great variability among individuals, which are modified by genetic and long-term lifestyle factors [4,5,6].

Fuelled by rapid urbanization, worldwide stroke occurrence is increasing in parallel with changes in lifestyle. Thus, women and men with a low-risk lifestyle (exercising daily, non-smoking, healthy diet, moderate ingestion of alcohol and being non-obese) had a significantly lower risk of stroke than individuals with known risk factors: sedentary lifestyle, arterial hypertension, high cholesterol, obesity, diabetes, heavy smokers, or alcoholism [7]. 

Comorbidities like diabetes, arterial hypertension or co-morbidity factors such as hypercholesterolemia, are common in elderly persons and are associated with a higher risk of stroke, increased mortality and disability [8]. Moreover, simultaneous presence of vascular diabetic complications-associated comorbidities like hypertension and chronic diabetes significantly increased the level of ischemic damage [9]. Therefore, ignoring comorbidities frequently associated with senescence, in experimental stroke research, could be one of the explanations for unsuccessful bench-to-bedside translation of neuroprotective strategies in aged subjects.

Young stroke patients may recover spontaneously after stroke. Stroke rehabilitation using physical training in older adults (<65 years) involving cardiorespiratory exercise, such as treadmill walking and cycling may lead to improved cognitive function, muscle strength, arm function, balance and gait [10,11].

## 3. The Translational Road Block in Stroke: A Failure to Account for Comorbidity?

Despite encouraging results from experiments with young animals, human stroke trials of neuroprotective factors aimed at limiting infarct expansion and promote tissue recovery after thrombolysis did not result in neuroprotection [12]. One possible explanation for this discrepancy between laboratory and clinical outcomes is the role that age plays in the recovery of the brain from insult [13].

Virtually all drug interventions that have been pre-clinically successful in experimental stroke have failed to translate this success to the clinical setting. We and others proposed that this is due to the failure of these pre-clinical studies to fully consider the aging and comorbidities for stroke that are present clinically [14,15,16,17,18,19,20,21]. It is quite possible that an intervention showing efficacy in abnormal animals may not be effective when co-morbidity exists. Moreover, a number of highly prevalent risk factors such as hypertension, diabetes and atherosclerosis are increasingly understood to act as “silent contributors” to neuroinflammation—not only establishing the condition as a central pathophysiological mechanism, but also constantly fuelling it.

## 4. Obesity as an Important Co-Morbidity for Ischemic Stroke

Stroke represents a potentially lethal condition with devastating effects for patients and their families. One of the most important risk factors for stroke is represented by obesity. At the present time, numerous studies conducted in the western world have recorded an “obesity epidemic” [22].

Obesity, characterized by a body mass index (BMI) of at least 30 kg/m^2^, is largely a consequence of fat-rich diet and physical inactivity and is associated with several risk factors including inflammation, metabolic syndrome, hypertension, diabetes and hypercholesterolaemia [8,22,23,24]. In particular, obesity increases the levels of inflammatory mediators such as C-reactive protein and interleukin-6, and chronic inflammation has been linked to worse stroke risk and outcome [21]. It has been shown that for obese patients, every 5 kg/m^2^ are associated with 40% increased mortality if the patient develops any type of stroke [8]. Furthermore, obese patients and those with metabolic syndrome are less likely to recanalise after thrombolytic therapy [25,26]. However, paradoxically, after cerebral ischemia, the young patients with an increased BMI were more likely to succomb to the acute event than the obese elderly patients who have a lower risk of death [22]. 

It has been proposed that obesity could represent a state of chronic inflammation that can be prevented to some extent by calorie restriction [27]. Indeed, it has been suggested that diminishing the expression of pro-inflammatory factors like inhibition of interleukin-1 (IL-1), a pro-inflammatory cytokine secreted in stroke patients, with the interleukin-1 receptor antagonist (IL-1Ra) after stroke may improve the post-stroke outcome [28]. The hypothesis has been tested in animal models whereby the IL-1 with IL-1Ra improves the odds of post-stroke outcome in overweight Wistar rats by increasing the number of post-stroke formed neurons from newly born neuroblasts [28].

## 5. Non-Pharmaceutical Approaches to Ischemic Stroke Prevention: Calorie Restriction 

Since obesity is a risk factor for stroke, there is an increasing interest in calorie restriction as a neuroprotective method in obese patients [27,29]. It has been proposed that calorie restriction could be an effective method to combat obesity-associated inflammation and oxidative stress in obese subjects by interfering with to the IKK/NF-κB signaling pathway [30,31,32].

Observational studies have shown a strong correlation between blood lipid levels and stroke [33]. In animal models, it could be shown that VEGF-induced angiogenesis is compromised by hyperlipidemia and provided an explanation of poor efficacy of angiogenic therapies in patients suffering from hyperlipidemia [34,35].

Recently, we reported that weight reduction, before ischemic stroke, in 20-month-old, overweight rats induced by caloric restriction led to an early re-gain of weight. The beneficial effects of calorie restriction in aged subjects extended to indices of behavioral recuperation in tests that require complex sensorimotor skills, cutaneous sensitivity and spatial memory [36]. 

## 6. Reduction of Brain Inflammation by Gaseous Hypothermia Applied for 48 h 

Hypothermia represents a viable alternative to drugs to achieve post-stroke neuroprotection. In animal models of focal ischemia, hypothermia often reduces infarct size. Methods to achieve whole body cooling include ice packing, exposure to alcohol, water mattresses, or convective air blankets. However, temperature variations caused by a redistribution of blood flow due to vasoconstriction of superficial vasculature has prompted the search for an alternative method to induce long-term, regulated lowering of whole body temperature by using drugs [37,38]. 

The concept of drug-induced cooling has been inspired by nature. Hibernating beer, for example, sleeps in a self-created hydrogen sulfide (H_2_S) environment, which results in slower metabolism and hence lower body temperature [39]. Hydrogen sulfide is a weak, reversible inhibitor of oxidative phosphorylation, that lowers body temperature in mice to 15 °C over 6 h at an ambient temperature of 13 °C [39].

Gaseous hypothermia, either confined to the head or including the entire body, has been used to reduce post-stroke inflammation by a reduction in the metabolic rate both in animal studies and in humans [40,41,42,43]. For example, stroke patients have been exposed for 6 to 24 h to mild hypothermia achieved either by intranasal cooling or whole-body cooling. However, the clinical benefits of hypothermia as assessed by the NIHSS (National Institutes of Health stroke scale) scores, are limited, especially in the long-term [44,45,46]. Nevertheless, mild hypothermia might reduce infarct volume as compared to non-hypothermic patients [47]. Additional beneficial effects of mild hypothermia in human subjects include a reduction in cerebral edema and improved clinical outcome [48,49].

Recently, clinical outcome after longer exposure to therapeutic hypothermia (30–35 °C, started within 6 h of symptom onset) is being assessed in a multicenter, phase 3, randomized trial (EuroHYP-1) on patients with cardiac arrest. In this study, patients are randomly assigned to either hypothermia and drugs or best drug treatment alone for acute ischemic stroke [50]. 

Using a modified version of this procedure, we previously showed that post-stroke exposure of 20-month-old rats to H_2_S-induced hypothermia for 48 h resulted in a 50% reduction in infarct volume without causing obvious neurological or physiological side-effects [51,52].

In another study, cooling the animals to 33–35 °C for 48 h started at 2.5 h after stroke has resulted in improved recovery of the contralateral limb impairment in food pellet retrieval and reduced the infarct volume by 40% [53].

## 7. Twenty Four Hours of Hypothermia Has Temporary Efficacy in Reducing Brain Infarction and Inflammation in Aged Rats

In preliminary studies, we reported that 48 h post-stroke exposures to H_2_S lowers whole body temperature to about 30 °C and acts neuroprotectively in 20-month-old animals [51,52]. However, in order to avoid the complications such as shivering, associated with longer exposure to hypothermia in humans, we asked if a 24 h exposure to H_2_S, would have the same neuroprotective efficacy as a 48 h exposure.

In clinical trials, hypothermia has been applied for 2–48 h with various beneficial effects [54,55]. Therefore, the optimal time range of exposure to hypothermia yielding the most efficacious neuroprotection is not clear. While most authors agree that mild hypothermia (i.e., hypothermia range 32–34 °C) provides optimal neuroprotection [38,56], the ideal hypothermic therapy in terms of depth and duration of hypothermia has to be established yet. Thus, short exposure (2–3 h) to postischemic hypothermia may appear quite effective with short survival times (less than 24 h). In an attempt to find an optimal therapeutic window in aged rats, animals were exposed for 24 h to an atmosphere containing 80 ppm hydrogen sulfide (H_2_S) and 19.5% O_2_, achieved by mixing room-air with H_2_S-balanced nitrogen at a flow rate of 3 L/min. After 2 h, the concentration of H_2_S was adjusted to 40 ppm. Metabolic rate was measured via carbon dioxide production and oxygen consumption using gas detectors. Under these conditions, the body temperature, measured by telemetry, dropped to 32 ± 0.5 °C and the arterial blood pressure, which was also measured telemetrically, dropped by 30%. During this time the animals did not eat or drink. After 24 h animals were returned to normal atmospheric conditions. The animals recovered immediately and did not show any signs of neurological or physiological deterioration. 

**Figure 1:**
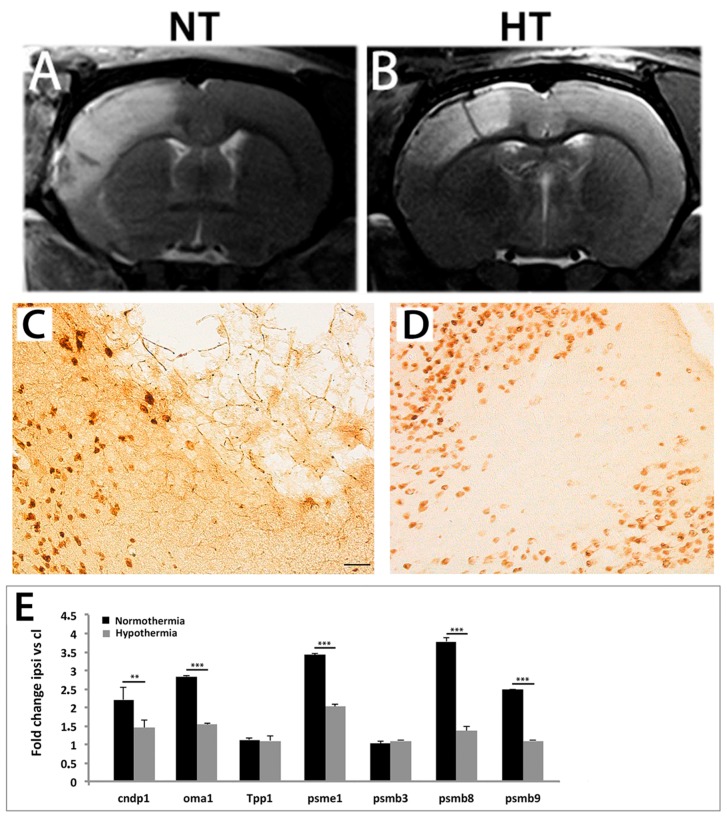
Forty eight hours of exposure to mild hypothermia resulted in a consistent reduction in infarct size. At 2 days post-stroke, the cortical lesion, as defined by the region of T2 hyperintensity, was reduced by the hypothermic treatment (**B**) as compared with the normothermic group (**A**) by ~42%. Tissue preservation in cooled animals ((**D**) vs. (**C**), controls) was associated with large decreases (fold-changes >2) in the transcripts coding for proteasome activator complex subunit 1 (psme), proteasome subunit beta type-8 and type 9 (psmb8, psmb9) as well as significant decreases in the mRNA coding for OMA1, a mitochondrial metalloendopeptidase, and to a lesser extent cndp1, a member of the M20 metalloprotease family, early after stroke (**E**). *Abbreviations*: NT, normothermia; HT, hypothermia. ** *p* < 0.01; *** *p* < 0.001, scale bar: 100 μm.

Twenty four hours of exposure to H_2_S-induced hypothermia did not result in consistent neuroprotection in post-stroke, 20-month-old animals. On the contrary, brain inflammation resumed at even higher pace so that by day 7, the infarct/edema volume exceeded that of controls suggesting that neuronal death was simply postponed. A longer exposure (48 h) was found to be more efficacious and resulted in a reduction in infarct volume and phagocytic activity of microglia at 2d and 14d post-stroke (Figure 1A–D).

Behaviorally, 24 h of exposure to hypothermia had a limited neuroprotective effect on the bilateral sensorimotor coordination (inclined plane) and asymmetry of limb placement (cylinder test). Body weight regulation was significantly improved in hypothermic animals post-stroke compared to animals kept at room temperature. Finally, a decreased number of phagocytic cells in hypothermia animals has been noted. At day 7 after stroke, 79% of cells expressing the polymorphonuclear cells (PMN) antigen also expressed ANXA1. At 14d, post-stroke ANXAA1 and ED1 co-localized in 47% of cells, in the infarct area.

## 8. Mechanisms of Hypothermia-Induced Protection against Ischemic Injury

A prerequisite for the successful translation of neuroprotective treatments from animal studies to clinical use is a more detailed understanding of the cellular and molecular mechanisms underlying for stroke treatment.

The mechanism underlying the beneficial effects of exposure to long-term hypothermia of post-stroke animals is not yet fully understood. The beneficial effects of hypothermia have been attributed to diminished excitotoxicity, neuroinflammation, apoptosis, free radical production, seizure activity, blood-brain barrier disruption, blood vessel leakage, and/or cerebral thermospooling [57]. Other neuroprotective mechanisms could include increased neurogenesis and an increased vascular density after stroke in the perilesional region of the injured, aged brains [58,59,60,61].

Normal aging is characterized by a chronic, low-grade, systemic inflammation caused by elevated levels of pro-inflammatory cytokines [62,63]. In the aged brain, inflammation becomes manifest by the chronic activation of perivascular and parenchymal macrophages/microglia expressing pro-inflammatory cytokines [64,65]. Therefore, it is not surprising that the age-associated pro-inflammatory state may contribute to the fulminant phagocytic activity of brain macrophages in the first 3 days post-stroke associated with the rapid development of the infarct in aged animals [66]. However, 48 h of exposure to mild hypothermia resulted in drastic downregulation of annexin A1 (ANXA1), a major pro-inflammatory protein that is upregulated after stroke and is expressed by polymorphonuclear cells in the perilesional cortex of post-ischemic, in the perilesional area of the aged rat brain.

At tissue level one clear effect of hypothermia was the preservation of the infarct core suggesting that the phagocytic activity of microglia was diminished in the animals kept under hypothermic conditions in the first two days post-stroke. Indeed, long term (48 h) of exposure to hypothermia caused a reduction in the transcriptional activity for mRNA coding for caspase 12, NF-κB and grp78 in the peri-infarcted region [51]. 

## 9. Post-Stroke Hypothermia Diminishes the Expression of Genes Coding for Proteases

An exacerbated upregulation of genes coding for proteases in the aged brain after stroke may be one reason for the severity of the damage as well as the brain’s resistance to neuroprotective therapies in the elderly. Prolonged exposure to H_2_S-induced hypothermia reduced infarct volume after stroke. As assessed by NeuN immunohistochemistry, the infarct core was better preserved in aged hypothermic animals compared to the tissue from aged normothermic animals, we hypothesized that the expression of genes coding for proteases might be attenuated by hypothermia. Indeed, proteasome inhibition has been shown to induce long-term neuroprotection after cerebral ischemia that is associated with reduced infarct size, improved functional neurological deficits, decreased blood–brain barrier breakdown and enhanced angioneurogenesis [67,68,69,70]. Proteasome subunit beta type-1, 8 and 9 are essential proteins that contribute to the complete assembly of the 20S proteasome complex that recognizes degradable proteins, including stroke-damaged proteins, for protein quality control purpose.

It has been hypothesized that long-term exposure to hypothermia may attenuate the expression of genes encoding proteases and improve the odds of tissue preservation and functional rehabilitation in post-stroke aged rats. To verify this hypothesis, post-stroke aged rats were exposed to two days of hypothermia and the expression levels of several proteasome-related genes were assessed by qRT-PCR. Indeed, it was found that two days of H_2_S-induced hypothermia significantly lowers the expression of genes coding for proteasomal proteins early after stroke, enhances microvascular density and improves indices of functional recovery at two weeks after stroke in 20-month-old rats [70] (Figure 1E). 

## 10. Post-Stroke Hypothermia Enhances Post-Stroke Angiogenesis in Aged Rats 

Mild hypothermia led to increased angiogenesis after focal ischemia in young adult rats [71,72]. However, in these studies neovascularization was detected using a non-specific marker, fluorescein isothiocyanate-dextran, which does not distinguish between old and newly formed blood vessels. Using an anti-collagen IV specific marker, it could be shown that hypothermia is also effective in enhancing the density of the newly formed vascular network in the formerly infarcted area of aged rats during the recovery phase after stroke [73,74,75].

## 11. Post-Stroke Hypothermia Does not Stimulate Neurogenesis in Post-Stroke Aged Rats 

Young rats exposed to hypothermia did show an increase in the endogenous repair capacity in the dentate gyrus as compared to normothermic animals [76,77,78]. Specifically, in these subjects, the number of newly formed neurons was higher in the hypothermic animals as compared to the normothermic ones. Along the same line, the number of new striatal neurons in post-stroke aged rats was similar to that in young rats despite a 50% decline in neurogenesis in the SVZ of older animals compared with young-adult animals [79]. There are very few studies relating hypothermia to neurogenesis. One study in adult rats showed that a 45 min exposure to hypothermia had no effect on neurogenesis in a rat model of forebrain ischemia [80].

An extremely important issue relates to the capacity of self-repair in the aged rodents brains. Recently, we reported that the number of large number of neuroblasts expressing DCX+ positive cells increased in the microenvironment of the SVZ of the injured, aged brain suggesting that the aged brain is still capable to mount a neurorestorative process [58,59]. Furthermore, inflammation as measured by Iba1-immunoreactive activated microglial cells, does not seem to impair the neurogenic response to the SVZ [59]. Hypothermia did not, however, increase the number of neuroblasts in the SVZ and infarct area, suggesting that factors, such deregulation in the induction of immediate early genes may block the beneficial effect of hypothermia on post-stroke neurogenesis.

Conclusions: Data from our laboratory and elsewhere indicate that the aged brain still has the capability to mount a cellular and molecular response to cerebral ischemia, but the timing of the cellular and genetic response to an overt injury such as stroke is dysregulated in the aged brain [81,82]. 

Furthermore, the use of animal models of stroke, which often ignore comorbidities frequently associated with senescence, could be one of the explanations for unsuccessful bench-to-bedside translation of neuroprotective strategies [83,84,85,86,87,88,89,90]. Major efforts are directed toward reducing post-stroke neuroinflammation which is the major cause of brain edema and mortality in the first week after the event in humans. A promising agent to reduce post-stroke neuroinflammation is hypothermia.
